# Gaps in Mental Health Care–Seeking Among Health Care Providers During the COVID-19 Pandemic — United States, September 2022–May 2023

**DOI:** 10.15585/mmwr.mm7402a1

**Published:** 2025-01-16

**Authors:** Anthony Papa, John P. Barile, Haomiao Jia, William W. Thompson, Rebecca J. Guerin

**Affiliations:** ^1^Department of Psychology, University of Hawaii at Manoa, Honolulu, Hawaii; ^2^Department of Biostatistics, Mailman School of Public Health and School of Nursing, Columbia University, New York, New York; ^3^Division of Viral Hepatitis, National Center for HIV/AIDS, Viral Hepatitis, STD, and TB Prevention, CDC; ^4^Western States Division, National Institute for Occupational Safety and Health, CDC.

**Summary**

**What is already known about this topic?**

Providing patient care during the COVID-19 pandemic has been associated with high levels of mental health symptoms among U.S. health care workers.

**What is added by this report?**

Among providers surveyed, 26% reported mental health symptoms at levels meeting diagnostic criteria during September 2022–May 2023; however, only 20% of providers sought mental health care during the preceding year. Support from supervisors reduced the effect of work stressors on mental health symptoms. The primary barriers to care-seeking were difficulty getting time off from work and concerns about confidentiality and cost.

**What are the implications for public health practice?**

Gaps in mental health care–seeking might be reduced by organizational and governmental efforts to reduce stigma, addressing concerns about confidentiality and licensing, and increasing supervisor training.

## Abstract

Health care workers experience substantial chronic stress, burnout, and mental distress, and the COVID-19 pandemic might have exacerbated these conditions. To identify ways to improve mental health care–seeking among this population, mental health symptoms, care-seeking, and self-reported barriers to seeking mental health care among U.S. health care providers during the pandemic were studied. During September 2022–May 2023, 2,603 primary care physicians, pediatricians, nurse practitioners, and physician assistants participated in a national Internet panel survey. Approximately one half (45.4%) of participants reported that they did not need mental health care, and only one in five (20.3%) had sought care. One quarter (25.6%) of providers reported mental distress severe enough to meet diagnostic criteria for psychopathology. Among these providers, only 38% reported seeking care; 20.1% indicated that they did not need care, despite severe symptoms. The average number of years in practice was lower for providers reporting care-seeking. Providers who identified as female were also more likely to report care-seeking. The most frequently reported barriers to care-seeking included difficulty getting time off from work, cost of care, and concerns about confidentiality. Increased pandemic-related work stressors were associated with increased symptom severity, but support from work supervisors mitigated these effects. Organizational human resources practices, supervisor training on managing employee stress, and public health messaging to normalize mental health care–seeking and its effects on licensing might help address gaps in provider care-seeking and improve patient outcomes.

## Introduction

Health care workers have long experienced high levels of chronic stress, burnout, and mental distress*; for some health care workers, these conditions were exacerbated by providing care during the COVID-19 pandemic (*1*). Two known and potentially interrelated factors affecting provider mental health are mental health care–seeking and social-emotional support from work supervisors. To help guide activities to increase mental health care–seeking among this population, this study examined the associations among self-reported mental health needs, care-seeking, factors associated with care-seeking, and perceived support from supervisors among a national sample of health care providers.

## Methods

### Data Source and Data Collection

Data were collected by Porter Novelli Public Services as part of its DocStyles survey from two Internet-delivered panel surveys of U.S. health care providers during September 9–November 3, 2022, and during March 17–May 15, 2023.^†^ Primary care physicians, pediatricians, nurse practitioners, and physician assistants (2,063)^§^ practicing for ≥3 years who completed at least one survey were included in this cross-sectional data analysis.^¶^ Mental health needs were evaluated using standardized questionnaires. Anxiety and depressive symptoms** experienced during the preceding 2 weeks were determined using the Generalized Anxiety Disorder-2 scale and Patient Health Questionnaire-2 depression scale (*2*). Posttraumatic stress because of the COVID-19 pandemic was measured using the *Diagnostic and Statistical Manual of Mental Disorders, Fifth Edition*–indexed Posttraumatic Stress Disorder (PTSD) Primary Care Screen (*3*). Overall mental well-being was measured as mentally unhealthy days using one item from the CDC Health-Related Quality of Life-4 survey asking how many of the previous 30 days respondents’ mental health was not good (*4*).

Participants selected work stressors experienced from a list derived from existing literature on COVID-19–related occupational stressors for nurses.^††^ Perceived social support from work supervisors was determined using a modified item from the 2017 CDC Behavioral Risk Factor Surveillance System survey.^§§^ Mental health care–seeking was determined by asking, “In the past year, have you seen a doctor or other health professional about any of your emotions, nerves, or mental health?” Responses included, “Yes, I saw someone,” “No, I haven’t seen anyone,” “No, I didn’t need emotional or mental care,” or “Prefer not to say.” Those who did not seek care could select as many of the seven barriers to care-seeking as applied to them.

### Data Analysis

Statistical differences among care-seeking groups were determined using two-sided *z-*tests of equality for proportions or *t*-tests for mean scores. Logistic regression was used to determine how supervisor support, as a moderating variable, influenced the association between work stress, as a dependent variable, and the likelihood of meeting diagnostic criteria for a mental disorder in separate models for anxiety, depression, and posttraumatic stress symptoms, as well as mentally unhealthy days, as dependent variables with no covariates. Analyses were completed using R (version 4.2.2; R Foundation).^¶¶^ This activity was reviewed by CDC, deemed not research, and was conducted consistent with applicable federal law and CDC policy.***

## Results

### Mental Health and Care-Seeking

Among 2603 surveyed healthcare workers, 667 (25.6%) reported levels of mental distress severe enough to meet diagnostic criteria for a mental disorder, but only 526 (20.3%) of respondents reported seeking mental health care (226 [43.0%] of these reported diagnostic levels of symptoms) (Table 1). Of providers who reported diagnostic levels of mental distress, 20.1% reported not needing care. The percentage of providers reporting mental distress meeting diagnostic levels was higher among those who did not seek care for their health needs than it was among those who reported not needing care, when measuring depression (38.3% versus 18.8%), anxiety (39.1% versus 16.4%), posttraumatic stress (34.5% versus 22.2%), and mentally unhealthy days per month (37.9% versus 14.6%). The proportion of providers reporting symptom severity indicating they met criteria for a mental disorder was similar for those who sought mental health care and those who had not sought this care. The percentage of providers reporting diagnostic levels of mental distress was lower for those who preferred not to report mental health care–seeking than for those reporting not needing care.

**TABLE 1:** Sample characteristics and mental health outcomes among a sample of health care providers, by health care–seeking — Porter Novelli DocStyles survey, United States, fall 2022 and spring 2023

Characteristic	No. (%)							
	Total sample N = 2,603 (100.0%)	Sought care n = 526 (20.3%)		Did not seek care n = 743 (28.7%)		Did not need care n = 1,179 (45.4%)	Preferred not to say n = 155 (6.0%)	
	Column % (95% CI)	Row % (95% CI)	p-values*	Row % (95% CI)	p-value^†^	Row % (95% CI)	Row % (95% CI)	p-value^†^
**Provider type**								
Pediatrician (n = 431)	**16.6 (15.2–18.0)**	24.4 (20.5–28.6)	0.081, <0.001	28.8 (24.6–33.2)	<0.001	41.1 (36.5–45.8)	5.8 (3.9–8.3)	<0.001
Physician assistant (n = 219)	**8.4 (7.4–9.5)**	27.9 (22.2–34.1)	0.858, <0.001	27.4 (21.8–33.6)	<0.001	40.6 (34.3–47.2)	4.1 (2.1–7.4)	<0.001
Primary care physician (n = 1,723)	**66.2 (64.4–68.0)**	16.4 (14.7–18.2)	<0.001, <0.001	29.6 (27.5–31.8)	<0.001	47.4 (45.1–49.8)	6.6 (5.5–7.9)	<0.001
Nurse practitioner (n = 230)	**8.8 (7.8–10.0)**	33.9 (28.0–40.2)	<0.001, 0.002	21.3 (16.4–26.9)	<0.001	41.7 (35.5–48.2)	3.0 (1.4–5.9)	<0.001
**Gender identification**								
Female (n = 1,072)	**41.2 (39.3–43.1)**	26.1 (23.6–28.8)	0.257, <0.001	29.0 (26.4–31.8)	<0.001	40.1 (37.2–43.1)	4.8 (3.6–6.2)	<0.001
Male (n = 1,499)	**57.6 (55.7–59.5)**	16.0 (14.2–17.9)	<0.001, <0.001	28.5 (26.2–30.8)	<0.001	49.2 (46.6–51.7)	6.3 (5.2–7.7)	<0.001
Other (n = 32)	**1.2 (0.9–1.7)**	18.8 (8.2–34.6)	0.144, <0.001	15.6 (6.2–30.9)	<0.001	37.5 (22.4–54.8)	28.1 (14.9–45.1)	0.023
**Exceeded established cut scores on mental health symptom measures indicating a high probability of meeting diagnostic criteria for psychopathology** ^§^								
Above any cut score	**25.6 (23.9 – 27.4)**	37.6 (33.8–41.5)	0.904, <0.001	37.3 (33.5–41.2)	<0.001	20.1 (17.1–23.5)	5.0 (3.5–7.0)	<0.001
Anxiety	**17.3 (15.9–18.8)**	38.4 (34.0–43.0)	0.812, <0.001	39.1 (34.7–43.7)	<0.001	16.4 (13.2–20.1)	6.0 (4.1–8.5)	0.001
Depression	**10.2 (9.1–11.4)**	35.0 (29.4–40.8)	0.219, <0.001	38.3 (32.7–44.3)	<0.001	18.8 (14.5–23.8)	7.9 (5.1–11.6)	0.001
Mentally unhealthy days	**11.1 (9.9–12.4)**	41.0 (35.2–47.0)	0.271, <0.001	37.9 (32.2–43.9)	<0.001	14.6 (10.7–19.2)	6.5 (4.0–10.0)	0.006
Posttraumatic stress	**6.6 (5.7–7.6)**	37.4 (30.4–44.8)	0.284, <0.001	34.5 (27.7–41.8)	<0.001	22.2 (16.5–28.9)	5.8 (3.0–10.1)	<0.001
**Other measures and characteristics**								
Anxiety score, mean**	**1.3 (1.3–1.4)**	2.1 (2.0–2.3)	<0.001, <0.001	1.7 (1.6–1.8)	<0.001	0.8 (0.7–0.8)	1.3 (1.1–1.6)	<0.001
Depression score, mean^††^	**0.9 (0.8–0.9)**	1.4 (1.3–1.5)	0.010, <0.001	1.2 (1.1–1.3)	<0.001	0.5 (0.4–0.5)	1.0 (0.8–1.2)	<0.001
No. of mentally unhealthy days, mean^¶^	**4.2 (3.9–4.5)**	7.8 (7.1–8.6)	<0.001, <0.001	5.4 (4.8–6.0)	<0.001	1.8 (1.6–2.1)	5.1 (3.5–6.7)	<0.001
No. of patients per week, mean	**104.6 (101.8–107.4)**	103.1 (96.7–109.5)	1.000, 1.000	103.6 (98.8–108.4)	1.00	105.9 (101.6–110.1)	105.0 (93.8–116.2)	1.00
No. of work stressors, mean^¶¶^	**8.3 (8.0–8.6)**	10.1 (9.6–10.7)	<0.001, <0.001	8.8 (8.4–9.3)	<0.001	7.0 (5.8–8.1)	7.4 (7.1–7.8)	0.971
Posttraumatic stress score, mean^§§^	**1.2 (1.1–1.2)**	1.7 (1.6–1.9)	<0.001, <0.001	1.4 (1.4–1.5)	<0.001	0.8 (0.7–0.9)	1.0 (0.8–1.2)	0.262
Supervisor social support score, mean***	**2.6 (2.6–2.7)**	2.8 (2.7–2.9)	0.262, 0.014	2.6 (2.5–2.7)	0.693	2.5 (2.4–2.6)	2.6 (2.4–2.8)	0.946
**No. of years practicing medicine, mean**	**15.3 (14.9–15.6)**	13.0 (12.3–13.7)	0.004, <0.001	14.9 (14.2–15.5)	<0.001	16.9 (16.4–17.5)	12.7 (11.5–13.9)	<0.001

* p-values reported are for comparisons with “Did not seek care” and “Did not need care,” respectively.

^†^ p-values reported are for comparisons with “Did not need care.”

^§^ Cut scores are empirically established scores that, if exceeded, demarcate the threshold when the probability for a person to meet the diagnostic criteria for a related psychiatric diagnosis is very high.

^¶^ Mean number of mentally unhealthy days in the previous month (range = 0–30). Scores ≥14 indicate a strong likelihood of having a mental health diagnosis.

** Mean Generalized Anxiety Disorder-2 total score; two items rated 0–3 (not at all to nearly every day) (range = 0–6). Scores ≥3 indicate a strong likelihood of having a mental health diagnosis.

^††^ Mean Patient Health Questionnaire-2 total score; two items rated 0–3 (not at all to nearly every day) (range = 0–6). Scores ≥3 indicate a strong likelihood of having a mental health diagnosis.

^§§^ Mean Posttraumatic Stress Disorder-Primary Care Screen total score where respondents indicated how many of the five core symptoms of posttraumatic stress they had experienced because of the pandemic (range = 0–5). Scores ≥4 indicate a strong likelihood of having a mental health diagnosis.

^¶¶^ Mean number of work stressors experienced during the pandemic (range = 0–14).

*** Mean score of social support from supervisors, rated on a scale ranging from 1–5 (never to always).

### Provider Characteristics Associated with Care-Seeking

Providers who reported they did not need care had been in practice the longest (median [IQR] = 13.0 years [15.0]). Fewer male providers sought care (16.0%) than did female providers (26.1%) or those identifying as other than male or female (18.8%). Primary care physicians, 68.7% of whom were male, reported the lowest prevalence of seeking care (16.4%). Nurse practitioners (33.9%; 81.7% female), physician assistants (27.9%; 68.5% female), and pediatricians (24.4%; 50.1% female) reported the highest prevalence of seeking care.

### Work Stressors and Barriers to Seeking Mental Health Care

The main work stressors reported by health care providers were extra stress at work (68.2%), burnout (58.9%), lack of adequate staffing (58.9%), higher workload or job demands (57.2%), fear of becoming ill with COVID-19 (55.6%), and COVID-19 misinformation (51.3%) (Table 2). Among respondents who did not seek care, the most frequently reported barrier was difficulty getting time off work, followed by concerns about confidentiality, cost, and being seen as weak.

**TABLE 2:** Work stressors and barriers to mental health care* reported by a sample of health care providers, by health care–seeking — Porter Novelli DocStyles survey, United States, fall 2022 and spring 2023

Work stressors/Barriers	% (95% CI)				
	Total N = 2,603 (100.0%)	Sought care n = 526 (20.3%)	Did not seek care n = 743 (28.7%)	Did not need care n = 1,179 (45.4%)	Preferred not to say n = 155 (6.0%)
**Work stressors**					
Burnout	**58.9 (57.0–60.7)**	75.7 (71.9–79.2)	63.4 (59.9–66.8)	48.8 (45.9–51.6)	56.8 (48.9–64.4)
COVID-19 misinformation	**51.3 (49.4–53.2)**	61.6 (57.4–65.7)	51.1 (47.6–54.7)	48.3 (45.5–51.2)	39.4 (31.9–47.2)
Extra stress at work	**68.2 (66.3–69.9)**	81.6 (78.1–84.7)	70.4 (67.0–73.6)	61.7 (58.9–64.5)	60.6 (52.8–68.1)
Fear of becoming ill with COVID-19	**55.6 (53.6–57.5)**	61.4 (57.2–65.5)	58.0 (54.4–61.5)	53.4 (50.6–56.3)	40.0 (32.5–47.8)
Fear of spreading COVID-19 to others	**49.3 (47.4–51.2)**	59.9 (55.7–64.0)	52.2 (48.6–55.8)	43.5 (40.7–46.4)	43.9 (36.2–51.7)
Higher workload or job demands	**57.2 (55.3–59.1)**	68.3 (64.2–72.1)	58.1 (54.6–61.7)	52.8 (50.0–55.7)	48.4 (40.6–56.2)
Insufficient capacity to give self-care	**34.2 (32.3–36.0)**	49.8 (45.5–54.1)	42.5 (39.0–46.1)	22.2 (19.9–24.7)	31.6 (24.7–39.2)
Isolation from family or friends	**41.5 (39.0–44.0)**	57.2 (51.4–62.9)	46.8 (42.2–51.4)	32.9 (29.6–36.4)	35.1 (26.1–44.9)
Lack of adequate staffing	**58.9 (57.0–60.7)**	66.9 (62.8–70.8)	61.0 (57.4–64.4)	55.4 (52.5–58.2)	47.7 (40.0–55.6)
Lack of beds for COVID-19 patients	**27.2 (25.5–28.9)**	31.6 (27.7–35.6)	29.1 (25.9–32.4)	25.2 (22.8–27.7)	18.7 (13.2–25.4)
Lack of clear guidance or treatment protocols	**45.1 (43.2–47.0)**	54.2 (49.9–58.4)	45.9 (42.3–49.5)	42.3 (39.5–45.2)	31.0 (24.1–38.5)
Lack of COVID-19 tests and timely results	**37.8 (36.0–39.7)**	43.7 (39.5–48.0)	36.2 (32.8–39.7)	37.6 (34.8–40.4)	27.1 (20.6–34.5)
Lack of manager concern for my well-being	**23.9 (22.3–25.6)**	31.2 (27.3–35.2)	27.7 (24.6–31.0)	18.3 (16.2–20.6)	23.2 (17.1–30.3)
Lack of personal protective equipment	**34.1 (32.3–35.9)**	39.9 (35.8–44.2)	36.7 (33.3–40.3)	30.8 (28.2–33.5)	26.5 (20.0–33.8)
Lack of supplies (e.g., for cleaning)	**32.5 (30.7–34.3)**	39.4 (35.2–43.6)	34.7 (31.4–38.2)	29.0 (26.5–31.6)	25.2 (18.8–32.4)
Longer shifts or work hours	**41.2 (39.3–43.1)**	47.5 (43.3–51.8)	46.7 (43.1–50.3)	34.9 (32.2–37.6)	41.9 (34.4–49.8)
My job was putting me at great risk	**45.2 (43.3–47.1)**	54.0 (49.7–58.2)	48.5 (44.9–52.0)	40.0 (37.3–42.9)	39.4 (31.9–47.2)
Need for constant awareness or vigilance	**44.5 (42.6–46.4)**	53.4 (49.2–57.7)	46.4 (42.9–50.0)	41.3 (38.5–44.1)	29.7 (22.9–37.2)
Shortages of equipment (e.g., ventilators)	**25.9 (24.2–27.6)**	30.4 (26.6–34.4)	28.5 (25.4–31.9)	23.1 (20.7–25.5)	18.7 (13.2–25.4)
Stigma from caring for COVID-19 patients	**17.3 (15.9–18.8)**	20.3 (17.1–23.9)	19.7 (16.9–22.6)	14.2 (12.3–16.2)	19.4 (13.7–26.1)
Barriers to seeking care*					
Care costs too much money	**—**	—	19.5 (16.8–22.5)	—	—
I am afraid of losing my job	**—**	—	6.5 (4.9–8.4)	—	—
I am worried about confidentiality	**—**	—	21.8 (18.9–24.9)	—	—
I had treatment before, and it didn’t help	**—**	—	5.0 (3.6–6.7)	—	—
It’s difficult to get time off work	**—**	—	44.4 (40.9–48.0)	—	—
I would be seen as weak	**—**	—	12.1 (9.9–14.6)	—	—
Other reason not listed	**—**	—	39.7 (36.2–43.3)	—	—

* Only among providers who did not seek care.

### Association of Outcomes with Supervisor Support

Each increase in the number of work stressors increased the odds of meeting diagnostic criteria for psychopathology by 9% for the anxiety scale (OR = 1.09; 95% CI = 1.06–1.12), 3% for the depression scale (OR = 1.03; 95% CI = 1.00–1.07), 35% for the posttraumatic stress scale (OR = 1.35; 95% CI = 1.29–1.41), and 12% for number of mentally unhealthy days (OR = 1.12; 95% CI = 1.08–1.17). However, the strength of association between the level of reported work stressors (low to high) and the likelihood of meeting diagnostic criteria for a mental health problem (anxiety, depression, and mentally unhealthy days) decreased as social support from supervisors increased (Figure). Similarly, among providers who did not seek mental health care, increased supervisor support reduced the association between increases in barriers to care-seeking and increased likelihood of meeting diagnostic criteria for a mental health problem. Supervisor support did not affect the probability of meeting a mental health diagnosis for posttraumatic stress.

**FIGURE:**
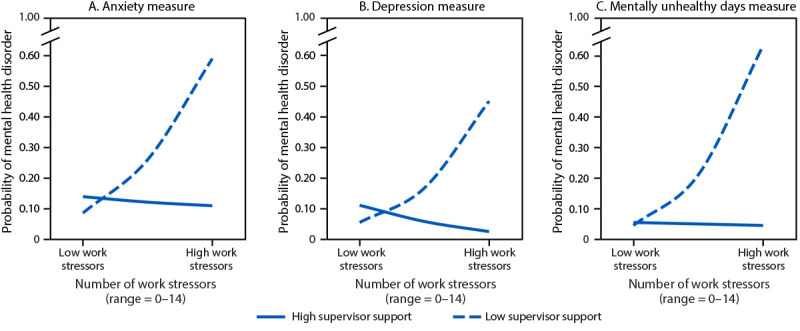
Interaction* of supervisor social support^†^ and number of work stressors^§^ on the probability of meeting diagnostic criteria for a mental health disorder based on responses to measures of anxiety^¶^ (A), depression** (B), and mentally unhealthy days^††^ (C) — Porter Novelli Services DocStyles Survey, United States, September 2022–May 2023^§§^ * Four separate logistic regressions were assessed to determine how supervisor support, as a moderating variable, influenced the association between work stress, as a dependent variable, and the likelihood of meeting diagnostic criteria for a mental disorder in separate models for anxiety, depression, and posttraumatic stress symptoms, as well as mentally unhealthy days as dependent variables with no covariates. ^†^ Supervisor social support was rated on a scale ranging from 1–5 (never to always). ^§^ Mean number of work stressors experienced during the pandemic (range = 0–14). ^¶^ Anxiety was measured using the Generalized Anxiety Disorder–2 total score; two items rated from 0–3 (not at all to nearly every day) (range = 0–6). Scores ≥3 indicate a strong likelihood of having a mental health diagnosis. ** Depression was measured using the Patient Health Questionnaire–2 total score; two items rated from 0–3 (not at all to nearly every day) (range = 0–6). Scores ≥3 indicate a strong likelihood of having a mental health diagnosis. ^††^ Mentally unhealthy days in the last month (range = 0–30). Scores ≥14 indicate a strong likelihood of having a mental health diagnosis. ^§§^ Supervisor support did not affect the probability of meeting a mental health diagnosis for posttraumatic stress.

## Discussion

During September 2022–May 2023, approximately one quarter of U.S. health care providers included in the survey sample reported mental distress severe enough to meet diagnostic criteria, of which less than 40% reported seeking mental health care, with approximately one in five providers reporting that they did not need care. Experiencing higher numbers of COVID-19–related work stressors was associated with high symptom severity for anxiety, depression, and general mental health, but support from work supervisors appeared to mitigate the effect of these factors on mental health, with an exception for PTSD. The percentage of providers who reported burnout as an ongoing stressor is consistent with estimates from a 2021 survey of U.S. physicians (62.8%) (*5*). Similar to studies that have found significant stigma regarding reporting difficulties in mental health among providers (*1*,*6*), 6% of respondents indicated they preferred not to report mental health care–seeking and reported lower levels of symptom severity than did those who indicated they did not need care, possibly demonstrating reticence in reporting care-seeking extended to reporting work stressors. Overall, the high levels of clinically relevant symptoms reported by U.S. health care providers in this study might indicate problems in current health care workforce readiness, especially given that 57.4% of providers self-reporting severe symptoms did not seek mental health care, or indicated that they did not need care, potentially affecting patient outcomes (*7*).

### Limitations

The findings in this report are subject to at least four limitations. First, the respondents opted into the survey, which limits the representativeness of the results. Second, retrospective reporting on previous experience might have been subject to recall bias. Third, although the survey was anonymous, self-reported symptom severity and care-seeking might be subject to social desirability bias. Finally, other relevant indicators of adjustment beyond mental health, such as substance abuse, were not included.

### Implications for Public Health Practice

Organizational and governmental interventions will likely reduce stigma among health care providers by normalizing and supporting mental health care–seeking (*8*) and addressing perceived negative consequences of seeking health care on medical licensing (*5*). Mental health awareness and self-care training could be made a requirement of continuing education for maintenance of board certification and licensure at state levels. A recent review of interventions and resources to address health care workers’ mental health (*9*) identified organizational approaches to addressing the needs and barriers identified in this study. These include leadership development on organizational practices and work conditions affecting provider well-being; mentoring and peer support programs; training in mindfulness, stress reduction, self-compassion, and interpersonal communication; and brief psychotherapy programming. However, broad organizational implementation is lacking (*10*) and often does not reach those in community practice. Recent activities by Lorna Breen Act grantees^†††^ funded by the Health Resources and Services Administration include identifying effective organizational strategies, such as training for managers to address health care worker mental health and burnout. The Impact Wellbeing campaign^§§§^ from the National Institute for Occupational Safety and Health promotes the removal of barriers and reducing stigma for seeking mental health services and might provide a road map for advancing health care worker well-being.

## Footnotes

*
https://doi.org/10.17226/25521

† Porter Novelli Public Services collected data as a part of its DocStyles survey using the global market research company SERMO’s Global Medical panel adhering to the Insights Association’s standards and codes of conduct. https://www.insightsassociation.org/Resources/Code-of-Standards

§ A total of 3,008 surveys were completed during fall 2022 and spring 2023 administrations. The fall 2022 survey (September 9–November 3, 2022) had a 66% response rate (1,505) and the spring 2023 survey (March 17–May 15, 2023) had a 64% response rate (1,503).

¶ Providers were matched by a unique survey identification number (no personally identifying information was included in the datasets) across the two data collection wave; 405 providers were identified as having completed both surveys, in which case, spring 2023 responses were not included in the analyses as these responses were further away from the pandemic’s peak. Thus, a total of 2,603 providers were included in the final analysis. Based on the number of questions completed, providers received a $50–$65 honorarium in the fall and $25–$39 in the spring. The questionnaires reported in this paper were the same for both DocStyles surveys. However, the spring survey had fewer questions overall, hence the difference in honorarium. Despite this, the response rates were comparable (66% versus 64%).

** All adjustment scales for anxiety, depression, posttraumatic stress, and mentally unhealthy days have established cutoff scores, demarcating a high probability of meeting diagnostic criteria for psychopathology.

††
https://www.icn.ch/system/files/documents/2020-09/Analysis_COVID-19%20survey%20feedback_14.09.2020%20EMBARGOED%20VERSION_0.pdf

§§ The question, “How often do you get the social and emotional support you need,” from the 2017 CDC Behavioral Risk Factor Surveillance System survey (https://www.cdc.gov/brfss/questionnaires/pdf-ques/2017_BRFSS_Pub_Ques_508_tagged.pdf) was modified to ask, “How often do you get the social and emotional support from your supervisor you need?” Respondents answered on a scale of 1–5 (never to always).

¶¶
https://www.r-project.org/

*** 45 C.F.R. part 46, 21 C.F.R. part 56; 42 U.S.C. Sect. 241(d); 5 U.S.C. Sect. 552a; 44 U.S.C. Sect. 3501 et seq.

††† Lorna Breen was an emergency department physician who died by suicide after not seeking mental health care for burnout during the COVID-19 pandemic for fear that receiving this care would jeopardize her medical career. https://drlornabreen.org/

§§§
https://www.cdc.gov/niosh/healthcare/impactwellbeing/about.html
